# Concentration of pistachio hull extract antioxidants using membrane separation and reduction of membrane fouling during process

**DOI:** 10.1002/fsn3.692

**Published:** 2018-08-06

**Authors:** Negin Seifzadeh, Mohammad Ali Sahari, Mohsen Barzegar, Hasan Ahmadi Gavlighi

**Affiliations:** ^1^ Department of Food Science and Technology Faculty of Agriculture Tarbiat Modares University Tehran Iran

**Keywords:** aqueous extraction, enzyme, pistachio green hull, polyphenols, ultrafiltration

## Abstract

The purpose of this study was to concentrate the polyphenolic compounds in pistachio hull extract as a rich source of natural antioxidants using the membrane process and the reduction of membrane fouling during the process. After the optimization of the membrane performance includes chamber pressure and stirring rate by monitoring flux and membrane fouling, pectinase and tannase enzymes were compared in order to reduce fouling. Pectinase showed a better potential in decreasing membranous fouling. Enzyme concentration was optimized, and treatment with 17.4 (U) was selected. The permeate obtained from optimized membrane condition and enzyme level treatment was enriched in total phenol (120.31 ± 0.35 mgGAE/g) and flavonoid (34.54 ± 0.09 mgCE/g), while the amount of anthocyanin was not remarkable.

## 1 INTRODUCTION

Synthetic antioxidants, such as butylated hydroxyanisole (BHA) and butylated hydroxytoluene (BHT), have been widely used as antioxidants in different food industries, but recently there have been some restrictions on the use of these compounds because of their carcinogenicity (Madhavi, Deshpande, & Salunkhe, 2017).

There is, therefore, a need for identifying alternative natural and safe sources of food antioxidants, especially of plant origin such as pistachio hull (Goli, Barzegar, & Sahari, 2005). Iran is one of the largest pistachio producers and exporters in the world. The total production of pistachio by Iran was about 249,000 tons in 2003 (Anonymous, 2003). Green hull is a major waste of the pistachio industry, which is estimated at an average of 60% of the production (Bohluli Ghaen, 2006). Pistachio green hull (PGH) is one of the richest sources of antioxidants, especially phenolic compounds (Chahed, China, & Daube, 2007; Goli et al., 2005; Mohammadi Moghaddam, Razavi, Malekzadegan, & Shaker Ardekani, 2009). Certain valuable studies have been published recently on the characterization of the phenolic profiles of pistachio green hull, but this topic still requires more detailed studies and deeper investigation.

In this study, the possibility of polyphenol extraction from pistachio hulls only by the use of water as a solvent and then concentrating them via the membrane system is assumed. Extraction of phenols from natural sources is usually conducted with chemical solvents (such as methanol and ethanol), whereas the corresponding fractions can be separated based on polarity. It is well known that many of these chemical solvents are toxic, highly flammable, detrimental to the environment and also costly in big scales; thus, in this research, water was used as an eco‐friendly and economic solvent (Płotka‐Wasylka, Rutkowska, Owczarek, Tobiszewski, & Namieśnik, 2017).

There is a possibility to concentrate phenolic compounds from the pistachio hull extract via membrane separation due to a difference between the molecular weights of the main ingredients of pistachio hull, such as polyphenolic compounds, protein, and pectin.

Membrane‐based processes, including UF^1^ , have been widely used in various industrial fields. It is among the conventional technologies that are utilized prior to or after the extraction process in order to separate macromolecules from smaller compounds in a physicochemical and nondestructive way (Liu et al., 2014). UF has been broadly used to treat fruit juices to obtain fractions enriched in phenolic. It has been recently reported that ultrafiltration and nanofiltration can be suited for the concentration and recovery of bioactive compounds from juices and byproducts (Avram et al., 2017; Conidi & Cassano, 2015; Conidi, Cassano, Caiazzo, & Drioli, 2017).

One of the most problematic issues that can interfere in the efficiency of ultrafiltration is membrane fouling. It is caused by the deposition of rejected material such as colloidal particles on the surface and within the pores of the membrane; results in flux decline and change in terms of membrane selectivity. Different methods to reduce membrane fouling include physical (e.g., ultrasound, sponge balls, back pulsing), biological (e.g., enzymatic treatment), and chemical (acids, alkalis, disinfectants, detergents) techniques (Saha & Balakrishnan, 2009). Thus, this problem can be overcome by the enzymatic treatment (by the usage of hemicellulases, phenol oxidase, and, in particular, pectinases) of the sample in which the colloidal particles are degraded before ultrafiltration (Kilara & Van Buren, 1995).

The main aim of this research is to concentrate and increase the amount of polyphenolic compounds in the pistachio green hull extract using the membrane system and to reduce membrane fouling during the process by the use of enzyme.

## 2 MATERIAL AND METHODS

### 2.1 Material

Pistachio green hulls (the Ahmadaghaei variety) were obtained from the Kerman Agricultural Research Center of Iran. The hulls were dried and ground, and then, a fraction that was sieved through a 10‐mesh sieve and retained on a 40‐mesh sieve was selected and stored in a freezer at −20°C until extraction. All the chemicals were analytical grade and obtained from the Sigma‐Aldrich Company Ltd. (Gillingham, UK) and Merck (Darmstadt, Germany) and were used without further purification.

### 2.2 Preparation of pistachio green hull extract

Extraction was carried out based on the optimized method by Rajaei, Barzegar, Mobarez, Sahari, and Esfahani (2010) and Goli et al. (2005). In this regard, water was used to extract phenolic compounds from the pistachio hull as a solvent. Therefore, 1 g of milled hull was subjected to the extraction, using a liquid‐to‐solid ratio of 1:15, during 8 hr and at 25°C (Goli et al., 2005; Rajaei et al., 2010).

### 2.3 Membrane operation system

Ultrafiltration of the pistachio hull extract was carried out in the batch dead‐end filtration plastic cell with a maximal capacity of 50 ml and an effective filtration surface area (A) of 13.4 cm^2^ (Amicon 8050, Merc Millipore, USA). A polysulfone membrane with a molecular weight CUT‐OFF SIZE (MWCO) of 100 kDa (GR‐40 pp, Alfa Laval) was used for filtration. The operational parameters, which may affect membrane separation performance include chamber pressure (1, 2.5, and 4 bar) and stirring rate (50, 150, and 250 rpm), were optimized. The pressure was prepared using a pump that was connected to the nitrogen container. The system also consisted of a magnetic stirrer, whose speed was controllable.

Prior to filtration, the membrane was washed by distilled water and then the filtration process of the pistachio hull extract was started. After filtration, distilled water was again filtered through the fouled membrane for the determination of membrane fouling. The most common way to evaluate membrane fouling included comparing water flux through origin and used membranes under the same operating conditions (Shi, Tal, Hankins, & Gitis, 2014).

(1) $$\%\;\text{Fouling}=\frac{{\text{flux}}_{1}-{\text{flux}}_{0}}{{\text{flux}}_{0}},$$

where flux_1_ is the water flux of the membrane after sample filtration at a given TMP and flux_0_ is the water flux through the virgin membrane at the same TMP (Shi et al., 2014). The filtrated flux was calculated using Equation (2):

(2) $$\text{Flux}=\frac{Q}{A*t},$$

where *Q* is the volume of the permeate (in L), *A* is the active membrane surface area (in m^2^), and *t* is the time taken for permeate filtration (in hr).

### 2.4 Enzymatic treatment of extract samples

In this regard, four levels of pectinase enzyme (5, 10, 15, and 25 μl/ml) were added to the 25 ml extract. Therefore, the treatments showed concentration levels (U) of 5.8, 11.6, 17.4, and 29.1, respectively. Then, the solutions were incubated at 50°C for 30 min in a thermoshaker system (120 rpm). The treatments were labeled P‐1, P‐2, P‐3, and P‐4, respectively. The control sample did not receive any enzyme treatment, but 5 ml of the sample was taken for further analysis and the remaining 20 ml was injected into the aforementioned membranous system and filtering process was continued till 15 ml of the extract passed through the membranous system. The flux of the pistachio hull aqueous extraction was monitored while filtering. The membranous fouling and the amounts of different phenol compounds, such as total phenol, total tannin, and antioxidant activity of extract, were measured in initial feed, permeate, and retentate through the following methods. Finally, the optimal dose of the enzyme was determined.

In order to compare the performance of the tannin enzyme with the performance of pectinase in terms of reducing the membrane fouling of this enzyme, 10 mg/g of dry material of the extract was added to 25 ml of the extract (concentration (U): 5/14). Then, the resulting mixture was incubated at 37°C for 2 hr in a thermoshaker system (120 rpm; T‐1 sample).

### 2.5 Determination of total phenolic compounds

The total phenolic compounds were determined using a modified version of the Folin–Ciocalteu colorimetric method (Waterhouse, 2001). To a mixture of 20 μl extract and 1.4 ml distilled water, 100 μl of Folin–Ciocalteu reagent (Sigma‐Aldrich, St‐Quentin Fallavier, France) was added. Then, 0.3 ml of Na_2_CO_3_ (75 g/L) was added and the mixture was vortexed. The sample was incubated for 30 min at 40°C. A UV–vis spectrophotometer (Seinco, Seoul, South Korea) was used for measuring absorbance. The resulting blue complex was then measured at 765 nm. Gallic acid (Sigma‐Aldrich, St‐Quentin Fallavier, France) was used for the calibration curve (Loginov, Boussetta, Lebovka, & Vorobiev, 2013).

### 2.6 Antioxidant activity

#### 2.6.1 DPPH**˙** assay

DPPH (2, 2‐diphenyl‐1‐picrylhydrazyl) radical‐scavenging activity was performed according to Blois (1958), with certain modifications. Different concentrations of the sample were mixed with 2.7 ml of 0.1 mM methanolic solution of DPPH radicals. The reaction mixture was vortexed and then incubated for 30 min at room temperature. The absorbance was read at 517 nm against a blank. The scavenging ability was calculated using the following equation:

(3) $$\begin{array}{cc} \text{Scavenging activity} & \;\;=\;\;(\left(A_{517}\;\text{control}\;\_\;A_{517}\;\text{sample}\right)/A_{517}\;\\ & \text{control})*100. \end{array}$$

The IC_50_ value is the effective concentration at which the DPPH radicals were scavenged by 50% and were obtained by interpolation from linear regression analysis (Hashemi, Aminlari, & Moosavi‐Nasab, 2014).

#### 2.6.2 ABTS**˙** ^+^ assay

ABTS**˙** (2, 2′‐azino‐bis (3‐ethylbenzothiazoline‐6‐sulfonic acid)) assay was carried out according to Arnao, Cano, and Acosta (2001) with certain modifications (Rajaee et al., 2010).

The radical cation solution was prepared by the reaction of 7.4 mM ABTS**˙** and 2.6 mM potassium persulfate solutions (in equal quantities), after 16 hr incubation at 22°C in a dark place. The ABTS^+^ solution was then diluted by methanol to obtain a solution with the absorbance of 1.00 ± 0.02 units at 734 nm. 200 ml of each sample was added to 4 ml of the ABTS^+^ solution, and after reacting for 2 hr in a dark place, the absorbance was measured at 734 nm. Ascorbic acid was used as standard and results were expressed in mg AA^2^ equivalents/mg phenolic.

### 2.7 Determination of total tannin

The total tannin was determined by measuring the nontannin phenols (NTP) and the precipitation of tannins using insoluble polyvinyl pyrrolidone (PVPP). 100 mg of insoluble polyvinylpyrrolidone was weighted, and 1 ml of distilled water and then 1 ml of extract were added and vortexed. The mixture was kept at 4°C for 15 min, vortexed again, and then centrifuged (3,000 × g) for 10 min; then, the supernatant was collected. The phenolic content of the supernatant was measured by the Folin–Ciocalteu reaction, and this was accepted as the NTP. Total tannins were calculated as the difference between total phenol and nontannin phenols (Makkar, Blummel, & Becker, 1995). Gallic acid was used as the standard.

### 2.8 Determination of total flavonoid content

The total flavonoid content was determined according to the colorimetric method described by Heimler, Vignolini, Dini, and Romani (2005). In 1.25 ml of distilled water, 250 μl of sample extract was mixed; then, 75 μl of 5% NaNO_2_ solution was added. After 6 min, 150 μl of 10% AlCl_3_·6H_2_O solution was added and allowed to stand for another 5 min before adding 0.5 ml of 1 M NaOH. The mixture was brought to 2.5 ml with distilled water and then vortexed. The absorbance was immediately read against the blank at 510 nm. Catechin was used as the standard.

### 2.9 Total anthocyanin content analysis

The pH differential method was used for anthocyanin determination (AOAC, 2003). This method was suitable in determining the total monomeric anthocyanin content based on structural changes in the anthocyanin chromophore between pH values of 1.0 and 4.5. Two portions (each portion was 0.2 ml) of pistachio hull extract were prepared. The first one was mixed with 3.8 ml of 0.025 M of potassium chloride buffer (pH value = 1.0) and the other portion mixed with 3.8 ml of 0.4 M sodium acetate buffer (pH value = 4.5). Absorbances were recorded at wavelengths of 510 and 700 nm for solutions at pH values of 1.0 and 4.5, respectively. The total anthocyanin content was expressed as cyanidin‐3‐glucoside (% w/w) equivalents as follows:

(4) $$\text{Total anthocyanin content}\;\left(\text{mg}/L\right)=\frac{A\;\times \;\text{MW}\left(\frac{g}{\text{mol}}\right)\;\times \;\text{DF}\;\times \;1,000}{\varepsilon \left(\frac{L}{\text{mol}}\text{cm}\right)\;\times \;L},$$

where *A* = [(A510 nm−A700 nm) pH = 1.0−(A510 nm−A700 nm) pH = 4.5]; MW of cyanidin‐3‐glucoside (cyd‐3‐glu (molecular weight) = 449.2 g/mol; DF = dilution factor; W = sample weight (mg); *L* = path length in cm; *ε* = 26,900 M extinction coefficient in L mol^−1^ cm^−1^ for cyd‐3‐glu.

### 2.10 Statistical analysis

Experimental data were analyzed using the analysis of variance (ANOVA), and significant differences among the means from triplicate analyses at *p <* 0.05 were determined by Duncan's test using the SPSS 19 software.

## 3 RESULTS AND DISCUSSION

About 40.7 g of aqueous extract was obtained per 100 g of green pistachio hull powder. The results of measuring the physicochemical characteristics of the aqueous extract of green pistachio hull are presented in Table 1, which conveys similar results on pistachio. Rajaei et al. (2010) reported that the total phenol of the PGH aqueous extract was 49.32 mg of GAE^3^ /g sample and EC_50_ of DPPH**˙** was 2.53 ± 0.02 μg phenolic/ml. In 2016, Barreca et al.^2016^ reported that the contents of total phenol, total flavonoids, and proanthocyanidins of the PGH methanol extract were 11.7 ± 0.48 (μM GAE), 0.688 ± 0.0197 (mg QE^4^ /g fresh weight), and 0.177 ± 0.004 (mg of cyanidin chloride equivalents/g of fresh water), respectively. This contents of the ethanol extract were 6.74 ± 0.42 (μM GAE), 0.341 ± 0.0062 (mg QE/g fresh weight), and 0.088 ± 0.001 (mg cyanidin chloride equivalents/g fresh water), respectively.

**Table 1:** Physicochemical characteristics of aqueous extract of green pistachio hull

Characteristics	Amount
Moisture (%)	97.33 ± 1.01
Protein (%)	0.24 ± 0.03
Pectin (mg/gDW^a^ pistachio hull)	45.30 ± 1.80
pH	4.62 ± 0.12
Total phenol (mg GAE/gDW pistachio hull)	94.79 ± 1.29
Total tannin (mg GAE/gDW pistachio hull)	29.09 ± 1.53
Flavonoid (mg CE^b^/gDW pistachio hull)	28.59 ± 0.54
Anthocyanin (mg/gDW pistachio hull)	0.12 ± 0.03
IC_50_ (ppm)	4.49 ± 0.14

*Notes*. ^a^Dry weight. ^b^Catechin equivalent.

As the first step, the prepared aqueous extract, was filtered using the 100 kD membrane under different conditions, including container pressure and mixer rotation, and (as mentioned in the previous section) its physiochemical parameters were calculated.

**Figure 1:**
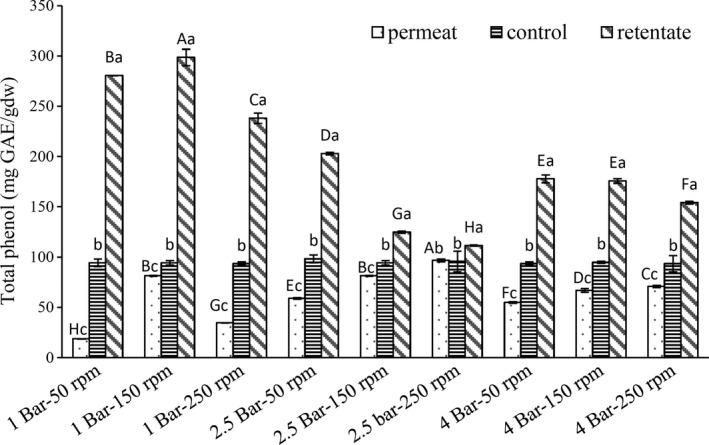
The total phenol of the fractions of membrane‐filtered crude extracts under different pressures and mixer speeds. Capital letters show significant difference among similar columns (*p* < 0.05). Small letters show significant difference among three columns of any treatment (*p* < 0.05)

As presented in Figure 1, among the membrane‐filtered samples, the highest and the lowest amount of total phenol was observed in the permeate and the retentate of the sample filtered under pressures of 2.5 and 1 bar, and mixer speeds of 250 and 50 rpm, respectively.

It is obvious that the level of phenolic compounds for all the samples in the retentate was more than the permeate after filtration; this could have happened due to membranous fouling during the process. Generally, phenolic compounds have lower molecular size than 100 kD, but owing to the presence of complicated compounds such as pectin in the pistachio hull extract, they can be trapped between pectin branches or they can also face difficulties in finding a way and passing through the membrane owing to its fouling.

In order to examine the membrane fouling behavior and to select the condition which generates the least fouling at the time of filtering the extract through the filter, the percentage of membranous fouling and the flux of samples through the membrane were measured using the methods mentioned in materials and methods. These results are presented in Table 2.

**Table 2:** Time of filtering, flux, and the percentage of membranous fouling of aqueous extract using 100‐kDa membrane under different pressures and mixer speeds

Treatments	Time (min)	Flux (ml/cm^2^ hr)	Fouling (%)
4 Bar‐250 rpm	69	0.97	60.14 ± 1.01^DE^
4 Bar‐150 rpm	75	0.90	65.84 ± 1.59^BC^
4 Bar‐50 rpm	107	0.63	68.56 ± 1.74^C^
2.5 bar‐250 rpm	68	0.99	56.86 ± 1.70^E^
2.5 Bar‐150 rpm	91	0.74	60.18 ± 2.18^DE^
2.5 Bar‐50 rpm	99	0.68	62.70 ± 0.19^CB^
1 Bar‐250 rpm	128	0.52	68.83 ± 3.41^B^
1 Bar‐150 rpm	130	0.52	69.17 ± 1.89^B^
1 Bar‐50 rpm	140	0.48	72.61 ± 1.48^A^

*Notes*. Different letters show significant (*p* < 0.05) difference in the columns (*p* < 0.05).

The highest level of membranous fouling was observed when the sample was filtered through the membrane under 1 bar of pressure and a mixing speed of 50 rpm. The best condition for filtering the extract, according to the maximum content of polyphenolic compounds and the antioxidant potential of permeate, was a pressure of 2.5 bar and a mixing speed of 250 rpm.

The aqueous extract of the green pistachio hull includes macromolecular polymeric compounds such as pectin, which could act as a membrane fouling agent. In 2017 Chaharbaghi, Khodaiyan, & Hosseini^2017^ reported the green pistachio hull as a valuable source of pectin. They showed that the maximum yield of pistachio green hull pectin obtained at the optimal conditions was 22.1 ± 0.5% (Chaharbaghi et al., 2017). In order to root out the problem of fouling in the current study, two tests were designed. The first step involved preparing a model system of the pectin solution, containing a definite amount of gallic acid. For this purpose, two pectin solutions (of 2 and 10 percent) were prepared, and a definite amount of gallic acid (almost equivalent to the extract of green pistachio hull) was added to them. To determine the percentage of membranous fouling and total phenol in the permeate and the retentate of filtration, the samples were filtered through a 100‐kDa membrane made of polysulfone. The results suggested that in the solution sample with low pectin, all the gallic acid was transferred to the permeate and no fouling was observed. In the case of the high content of the pectin solution, membranous fouling was observed and the retentate had a higher level of gallic acid than the permeate.

In the second test, 96% ethanol (3:1 v/v), pectinase enzyme, and tannase enzyme were used for the sedimentation and the decomposition of pectin as well as the decomposition of tannin (as a large molecule that can play a role in the fouling). After filtering the treated samples through the membrane, the total phenol contents of the permeate and the retentate were compared with the content of the sample (Table 3).

**Table 3:** Comparison between the effect of pectinase and tannase enzymes on membrane fouling needed time (in min) for passing definite amounts of sample through the membrane and the total phenol of the pistachio green hull extract

	Concentration (ppm)	Total phenol (mg GAE/gDW)				
		Retentate	Permeate	Fouling (%)	Time (min)	Fouling reduction (%)
Control	0	253.28 ± 0.53^A^	46.51 ± 0.70^C^	56.86 ± 1.70^A^	68	–
Pectinase	10	145.30 ± 7.81^C^	82.38 ± 4.46^A^	50.11 ± 0.13^C^	56	21.53 ± 0.38^A^
Tannase	10	168.56 ± 6.31^B^	73.43 ± 3.89^B^	53.27 ± 1.25^B^	60	16.51 ± 0.63^B^

*Notes*. DW: dry weight; GAE: Gallic acid equivalent.

Different letters show significant difference in the columns (*p* < 0.05).

The results suggest that pectinase is a more effective enzyme for reducing membranous fouling in comparison with tannase enzyme. This is probably due to the largeness of pectin molecules and their derivation from the pistachio extract. The total phenol in the sample treated with alcohol was reduced in comparison with the control sample. It seems that the underlying reason for this issue might be the concurrent deposition of phenolic compounds stuck between pectin branches and pectin molecules.

Based on the obtained results, one could conclude that the treatment of the extract by pectinase enzyme could be influential in reducing membranous fouling during the filtration process. Moreover, the concentration of the pectinase enzyme was optimized. In this regard, the effect of the pectinase enzyme of four concentrations was compared with the control sample. The physiochemical characteristics of extracts treated with different enzyme concentrations were determined after passing them through a 100‐kDa membrane and under‐optimized conditions (2.5 bar pressure; 250 rpm mixing).

**Figure 2:**
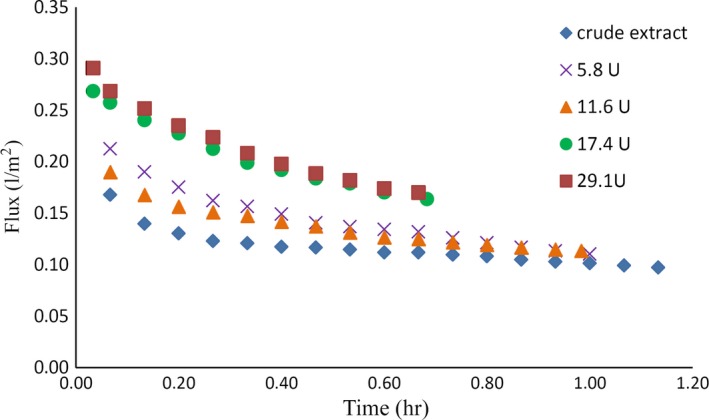
Flux of an aqueous extract treated with different pectinase concentration using a 100‐KDa membrane

As shown in Tables 4 and 5, and Figure 2, the use of any concentration of the pectinase enzyme leads to a reduction of membrane fouling and reduced time necessary for passing a definite volume of permeate, better transmission of antioxidant compounds across the membrane, and their concentration in the permeate section. As the concentration of enzyme increased from 5.8 to 29.1 (U) (5–15 ppm), the concentration of phenolic compounds in the permeate part increased, but the amount of these compounds and the antioxidant potential of the permeate part did not show any significant difference (*p <* 0.05) for 17.4 and 29.1 (U) concentrations. In addition, no statistically significant difference (*p < *0.05) was observed in terms of the required duration for transmitting a definite volume of permeate across the membrane and the extract flux between the enzyme concentrations of 5.8 and 29.1 (U). Considering the obtained results and the economic aspects of using a commercial level of enzyme, the optimal concentration of the enzyme required a reduction in the fouling of the membrane to 17.4 (U).

(5) In fact, using the equationR=(a−b/a)∗100,

**Table 4:** Characteristics of the fractions of membrane‐filtered samples treated with different pectinase concentrations

Pectinase concentration (U)	Total phenol (mg/gDW pistachio hull)			Total tannin (mg/gDW pistachio hull)			Total flavonoid (mg/gDW pistachio hull)		
	Sample	Retentate	Permeate	Sample	Retentate	Permeate	Sample	Retentate	Permeate
Control (0)	99.30 ± 0.52^Bc^	253.28 ± 0.53^Aa^	46.51 ± 0.70^DC^	29.09 ± 1.53^Bc^	66.48 ± 1.15^Aa^	10.72 ± 1.96^Db^	27.98 ± 0.08^ABb^	77.20 ± 0.71^Aa^	13.93 ± 0.14^Ec^
5.8	101.53 ± 0.87^ABb^	202.20 ± 4.23^Bc^	71.24 ± 5.55^Cc^	30.43 ± 0.41A^Bb^	67.49 ± 0.94^Aa^	10.10 ± 0.54^CDc^	27.99 ± 0.31^ABb^	68.22 ± 0.30^Bc^	14.52 ± 0.03 ^Da^
11.6	102.09 ± 0.73^Ab^	145.30 ± 7.81^Cc^	82.38 ± 4.46^Bc^	31.02 ± 0.31^Ab^	59.25 ± 0.71^Ba^	13.09 ± 2.00^Bc^	27.76 ± 0.38^Bb^	55.81 ± 1.61^Cc^	18.45 ± 0.12^Ca^
17.4	101.19 ± 1.58^ABb^	54.26 ± 1.35 ^Da^	120.31 ± 0.35^Aa^	29.59 ± 0.15^Bb^	53.41 ± 3.64^Ca^	16.64 ± 0.70^Ac^	27.51 ± 0.17^Bb^	21.96 ± 0.57 ^Da^	34.54 ± 0.09^Bc^
29.1	101.29 ± 2.65^ABb^	54.11 ± 1.21 ^Da^	119.58 ± 0.86^Aa^	29.01 ± 0.21^Bb^	52.96 ± 2.64^Ca^	16.93 ± 0.75^Ac^	28.65 ± 0.69^Ab^	21.18 ± 0.11 ^Da^	34.95 ± 0.06^Ac^

*Notes*. DW: dry weight.

Capital letters show significant difference in the columns (*p* < 0.05). Small letters show significant difference in each characteristic of fractions of membrane‐filtered sample (*p* < 0.05).

**Table 5:** Antioxidant activity of the fractions of membrane‐filtered samples treated with different pectinase concentrations

Pectinase concentration (U)	ABTS^+^ (gAAE/g phenolic)			IC_50_ (ppm)		
	Sample	Retentate	Permeate	Sample	Retentate	Permeate
Control (0)	6.12 ± 0.13^Aa^	5.23 ± 0.21^Ab^	4.23 ± 0.12^Cc^	4.49 ± 0.14^Aa^	4.27 ± 0.00^Ab^	4.56 ± 0.08^Ac^
5.8	6.24 ± 0.12^Aa^	5.41 ± 0.09^ABb^	6.17 ± 0.10^Ba^	4.52 ± 0.06^Ab^	4.72 ± 0.03^Ba^	3.92 ± 0.13^Bc^
11.6	6.34 ± 0.21^Aa^	5.87 ± 0.10^Bb^	6.34 ± 0.19^Ba^	4.53 ± 0.08^Ab^	5.82 ± 0.04^Cc^	3.79 ± 0.13^Ba^
17.4	6.25 ± 0.23^Ab^	3.65 ± 0.18^Cc^	8.35 ± 0.15^Aa^	4.47 ± 0.12^Ab^	6.36 ± 0.05 ^Da^	2.38 ± 0.10^Cc^
29.1	6.28 ± 0.18^Ab^	3.45 ± 0.13^Cc^	8.51 ± 0.14^Aa^	4.50 ± 0.05^Ab^	6.57 ± 0.22^Ea^	2.33 ± 0.19^Cc^

*Notes*. AAE: Ascorbic acid equivalent.

Capital letters show significant difference in the columns (*p* < 0.05). Small letters show significant difference in each characteristic of fractions of membrane‐ filtered sample (*p < *0.05).

where *R* denotes reduced fouling, *a* is the percentage of membranous fouling after transmitting control sample, and *b* is the membrane fouling after transmitting the sample treated with 17.4 (U) of pectinase enzyme, and it is observed that 17.4 (U) of the pectinase enzyme could reduce polysulfone membrane fouling with MWCO 100 kD, up to 44.86 percent (Table 6).

**Table 6:** Time of filtering and the percentage of membranous fouling of the aqueous extract treated with different pectinase concentrations using a 100‐kDa membrane

Enz. concentration (U)	Fouling (%)	Time (min)
0	56.86 ± 1.70^A^	68
5.8	54.99 ± 0.42^B^	60
11.6	50.11 ± 0.13^C^	56
17.4	31.35 ± 0.36^D^	41
29.1	29.73 ± 1.34^E^	40

*Notes*. Different letters show significant difference in the columns (*p* < 0.05).

Conidi et al., in 2017, investigated the separation of phenolic compounds from pomegranate juice using ultrafiltration and nanofiltration membranes. They compared four commercial flat‐sheet membranes with nominal MWCO ranging from 1,000 to 4,000 Da. First, they clarified the pomegranate juice using a cellulose triacetate UF membrane module. All the membranes were effective in concentrating phenolic compounds in the retentate and sugars in the permeate parts. MPF‐36 showed the highest retention value and increased the total phenol in the retentate from 2,457.5 ± 15.3 (mg GAE/L) in the clarified juice to 2,704.4 ± 14.1 (mg GAE/L). They used another completing filtration step with the Desgal GK membrane in order to increase the concentration of the phenolic compound in the retentate stream.

**Figure 3:**
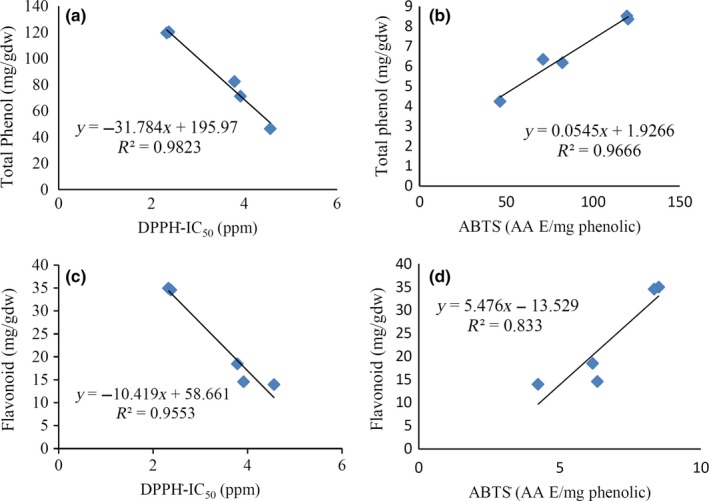
Linear regression between the total phenolic content (mg GAE/gdw) and (a) antioxidant activity by DPPH **˙** assay and (b) ABTS **˙** ^+^ assay and linear regression between the total flavonoid (mg CAE/gdw) and (c) antioxidant activity by DPPH**˙** assay and (d) ABTS **˙** ^+^ assay in the permeate of an enzyme‐treated filtered sample in the condition of 2.5 bar pressure and 250 rpm speed

Linear regression between the antioxidant capacity (DPPH**˙** and ABTS^+^) and total phenolic content and total flavonoid content in the permeate of the enzyme‐treated sample, in optimal conditions, is shown in Figure 3. The correlation coefficients of total phenols with DPPH and ABTS^+^ were 0.982 and 0.967, while the correlation coefficients of total flavonoids with DPPH and ABTS assays were 0.955 and 0.833, respectively. It was observed that the radical‐scavenging activity of the permeate of enzyme‐treated extract showed a better correlation with total phenols rather than flavonoids. It could be estimated that in the pistachio hull aqueous extract, the nonflavonoid phenols such as phenolic acids are more responsible for antioxidant activity than flavonoids. Kumar, Sandhir, and Ojha (2014) reported strong correlations between total phenol and antioxidant activity (DPPH**˙** and ABTṠ+) in *Lantana camara* leaves.

In 2012, Koseoglu‐Imer, Dizge, & Koyuncu^2012^ used different concentrations of the protease enzyme. By the immobilization of the enzyme over a membrane of cellulose acetate and a size of 100 kD (MWCO), they reduced the level of fouling by the proteins. The enzyme showed desirable performance in filtration. Maktouf et al., in 2014, treated lemon juice with *Penicillium occitanis* pectinase at various enzyme concentrations (0–1,200 U/L), temperatures (25–50°C), and times (0–90 min). Their application led to the reduction of viscosity and turbidity. These enzymatic treatments were followed by ultrafiltration (MWCO size of 15 kDa), which resulted in a higher permeate flux and a higher juice quality as well as lower microbiological contamination. Results suggested that enzymatic treatment, coupled with ultrafiltration, could be used for the production of lemon juice with high commercial value.

**Figure 4:**
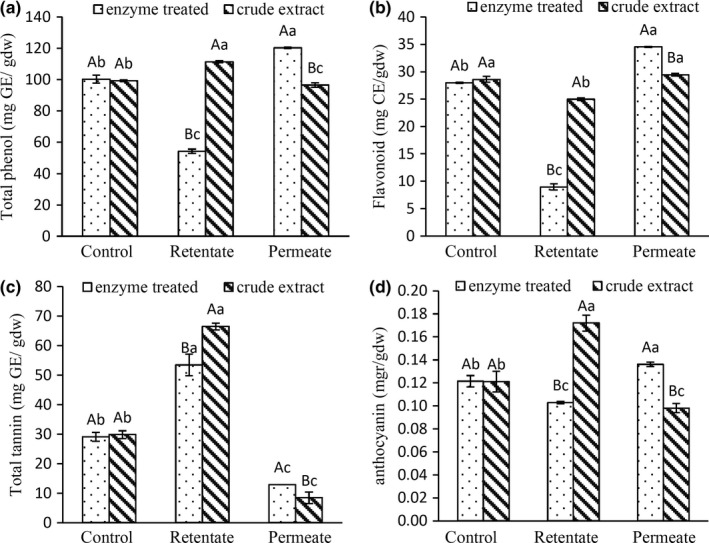
Comparison between the (a) total phenol, (b) total flavonoid, (c) tannin and (d) anthocyanin of control, retentate and permeate of pectinase enzyme‐treated and crude extracts, filtered in optimal condition. Capital letters show significant difference among similar columns (*p* < 0.05). Small letters show significant difference among three columns of any treatment (*p* < 0.05)

The chemical characteristics of control, permeate and retentate of membrane treated samples in the optimized condition (2.5 bar, 250 rpm), are shown in Figure 4, in order to have a bright comparison between enzyme‐treated and crude extracts.

As shown in Figure 4a, the permeate of pectinase treated extract in optimized concentration (17.4 U) has higher amount of phenolic compounds (121.31 ± 0.35 mg GAE/gDW) in comparison with the permeate of the crude extract (96.56 ± 1.39 mg GAE/gDW). In the crude extract, the membrane was not able to concentrate phenolic compounds in the permeate, due to its fouling by high molecular weight compounds such as pectin. Phenols are the most responsible compounds for pistachio hull extract antioxidant activity. Plenty of researches reported a remarkable antioxidant potential of pistachio hull extract (Barreca et al., 2016; Rajaee et al.*,* 2010; Goli et al., 2005). Gallic acid, β‐glucogallin, galloyl quinic acid, 4‐hydroxybenzoic acid, eriodictyol‐7‐o‐glucoside, quercetin‐3‐o‐rutinoside, isorhamnetin‐3‐o‐rutinoside, and catechin were reported as the most abundant compounds among phenolic compounds of pistachio hull methanolic extract (Barreca et al., 2016; Erşan et al., 2016).

The results of measuring the flavonoid compounds shown in Figure 4b suggest that a significant level of all the phenolic compounds (about 28 percent) in an aqueous extract of green pistachio hull included flavonoid compounds. Similar results were reported by Barreca et al. (2016). The amount of total flavonoid in the permeate of enzyme‐treated sample was increased from 29.09 ± 1.53 to 34.54 ± 0.09 mg CE/gDW after passing through the membrane, while the amount of flavonoids in the permeate of crude extract does not show a significant difference with the control sample.

The total tannin of membrane‐filtered samples as presented in Figure 4c showed that the total tannin in the aqueous extract of green pistachio hull constitutes about 56 percent of the total phenol compounds. Tannins have higher molecular weight than phenol compounds and they might play a role in membranous fouling. It should be noted that tannins were concentrated in the retentate for both the enzyme‐treated and crude extracts. Although the major part of tannin eliminated with 100‐kDa membrane in the retentate part, in the enzyme‐treated extract, more of the tannin compounds passed through the membrane.Tannin compounds can be trapped in the pectin branches and intensified membrane fouling. After pectin decomposition by the pectinase enzyme, probably these compounds are released from the branches of pectin and smaller molecules pass through the membrane.

Figure 4d shows that very low amounts of anthocyanin compounds in the aqueous extract of green pistachio hull; thus, they cannot have an impressive effect on the antioxidant activity of pistachio hull aqueous extract. The permeate resulting from the enzyme‐treated filtered sample had the higher level of anthocyanin compounds in comparison with the un‐filtered control extract, while in crude‐filtered extract, the major amount of anthocyanins concentrated in the retentate part.

**Figure 5:**
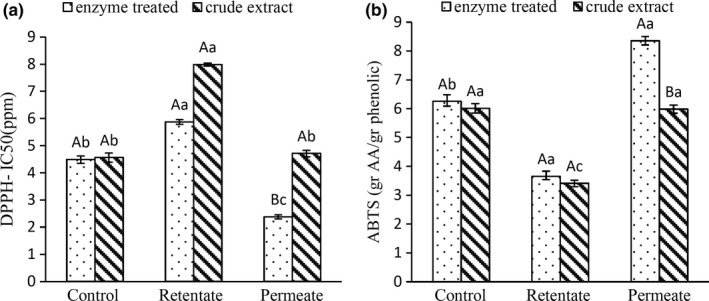
Comparison between the antioxidant activity of control, retentate and permeate of pectinase enzyme‐treated and crude extracts, filtered in optimal condition, expressed in (a) DPPH assay and (b) ABTS assay. Capital letters show significant differences among similar columns (*p* < 0.05). Small letters show significant differences among three columns of treatments (*p* < 0.05)

The antioxidant activity of enzyme‐treated and crude extracts, expressed in DPPH**˙** and ABTS**˙** ^+^ assays, is shown in Figure 5. Unstable free radical of DPPH is scavenged by antioxidants, as a result of electron donating and changes to a stable radical which has absorption at 517 nm. IC_50_ shows an effective concentration of extract needed for inhibiting 50 percent of free DPPH**˙** radicals (Barreca et al., 2016; Goli et al., 2005). For the determination of IC_50_, the best line of different concentration of the extract was used. In this regard, Figure 5a shows that 2.38 ± 0.8 ppm of the permeate of enzyme‐treated filtered extract of green pistachio hull is needed for controlling 50 percent of the free DPPH**˙** radicals, which demonstrates the excellent antioxidant activity, higher than the permeate of crude‐filtered extract. Although the retentate of crude‐filtered extract has a higher level of phenolic compounds from control and permeate, it shows a less antioxidant activity, probably due to the pro‐oxidant activity of its impurities. It can also suggest that the type of phenolic compounds is more effective on antioxidant activities than its quantity (Rajaee et al., 2010).

The radical cation‐scavenging activities of crude‐ and enzyme‐treated extracts, expressed as ascorbic acid equivalents, are shown in Figure 5b. ABTS^+^‐scavenging capacity of all samples meet the DPPH**˙** assay results and demonstrates the highest radical cation‐scavenging activity of the permeate of enzyme‐treated extract (8.35 ± 0.15 gAAE/g phenolic). ABTS^+^‐scavenging capacity of the permeate of crude extract was approximately equal to the control sample (unfiltered crude extract).

The results suggest that after treating the pistachio hull aqueous extract with the optimal level of pectinase enzyme, more amounts of phenolic compounds pass through the membrane due to the pectin decomposition and membrane fouling reduction and membrane concentration leads to separate a permeate part, rich in phenolic compounds and antioxidant activity.

## 4 CONCLUSION

The aqueous extract of pistachio green hull is a rich source of polyphenol compounds with antioxidant properties. The use of the polysulfonic membrane with 100 MWCO is an effective way to increase the concentration of the polyphenol compounds in the extract. To achieve this goal, conditions of the membrane process (pressure and agitation) were optimized and the best performance of the membrane was determined at a pressure of 2.5 bar and an agitation of 250 rpm. However, due to the presence of high molecular weight compounds, such as pectin in the extract, the membrane underwent fouling. Treating pistachio hull extract by the pectinase enzyme was found as an effective solution to reduce fouling. The proper concentration of enzyme was optimized and using 17.4 (U) of the enzyme showed an increase in the concentration of polyphenol compounds from 99.30 to 120.31 (mg/gDW), while it reduced membrane fouling up to 44.86% in comparison to the untreated extract.

## CONFLICT OF INTEREST

None declared.

## ETHICAL STATEMENTS

This study does not involve any human or animal testing.

## Endnotes

1 Ultra‐filtration.

2 Ascorbic acid.

3 Gallic acid equivalent.

4 Quercetin equivalent.
